# A Simulation-Based Diagnostic Stewardship Framework for Imaging Utilization in Primary Care: A Model Using 100 Common Clinical Conditions

**DOI:** 10.3390/diagnostics16142162

**Published:** 2026-07-10

**Authors:** Betül Tiryaki Baştuğ, Çağnur Elpen Kodaz, Sevil Akbulut Zencirci

**Affiliations:** 1 Department of Radiology, Faculty of Medicine, Şeyh Edebali University, 1100 Bilecik, Turkey; 2 Department of Family Medicine, Şeyh Edebali University, 1100 Bilecik, Turkey; cagnur.kodaz@bilecik.edu.tr; 3 Department of Public Health, Faculty of Medicine, Seyh Edebali University, 1100 Bilecik, Turkey; sevil.zencirci@bilecik.edu.tr

**Keywords:** primary care, diagnostic stewardship, imaging utilization, simulation modeling, radiation exposure, incidental findings, healthcare resource optimization, diagnostic decision-making, health systems efficiency, medical imaging policy

## Abstract

**Background:** The increasing utilization of diagnostic imaging has raised concerns regarding imaging overuse, unnecessary radiation exposure, and downstream diagnostic cascades. Because primary care physicians serve as the first point of contact for most patients, diagnostic decisions made in primary care may substantially influence healthcare resource utilization at the system level. This study aimed to develop and evaluate a conceptual diagnostic stewardship framework for primary care using a simulation-based modeling approach. **Methods:** A synthetic dataset consisting of 100 common primary care conditions was developed across ten clinical domains. Model parameters, imaging utilization probabilities, and diagnostic pathway assumptions were derived from literature-informed estimates and multidisciplinary expert judgment. Each condition was assigned diagnostic attributes including World Health Organization age group classification, commonly requested laboratory tests, preferred imaging modality, and imaging necessity classification (Class A: imaging usually unnecessary; Class B: conditional imaging; Class C: imaging usually required). Using this dataset, a simulation model representing one million hypothetical primary care visits was constructed. Imaging utilization, modality distribution, radiation burden index, incidental diagnostic cascades, and a relative diagnostic resource utilization index were estimated under a baseline diagnostic scenario and a framework-guided diagnostic stewardship scenario. **Results:** In the baseline scenario, the model generated 412,000 imaging examinations across one million simulated visits (41.2% imaging rate). Within the simulation model, application of the framework was associated with an estimated reduction in imaging examinations to 258,000, corresponding to a 37% reduction in imaging utilization. The estimated population-level radiation burden index decreased from 285,000 to 179,000 units, representing a 37% reduction in radiation exposure. The number of incidental diagnostic cascades decreased from 48,200 to 29,700 events, while the relative diagnostic resource utilization index decreased from 2,480,000 to 1,690,000 units. Sensitivity analyses confirmed the robustness of these findings across alternative model assumptions. **Conclusions:** Within the assumptions of this simulation model, the proposed diagnostic stewardship framework generated modeled reductions in imaging utilization, radiation burden, and downstream diagnostic consequences. These findings illustrate the potential impact of structured diagnostic stewardship strategies and provide a hypothesis-generating basis for future validation using real-world clinical data.

## 1. Introduction

Primary care represents the most accessible and frequently utilized component of healthcare systems worldwide. As the first point of contact for patients, family physicians manage a wide spectrum of acute, chronic, and preventive conditions while also serving as gatekeepers for further diagnostic testing, including laboratory investigations and imaging. Consequently, diagnostic decisions made in primary care have important implications not only for individual patient management but also for healthcare resource utilization and system efficiency [1,2].

In recent years, the use of diagnostic imaging has increased substantially. This trend has been driven by technological advances, expanding clinical indications, defensive medical practices, and growing patient expectations. Although imaging plays a central role in modern diagnostics, concerns have emerged regarding its potential overuse. Unnecessary imaging may expose patients to avoidable radiation, increase healthcare costs, and lead to incidental findings that trigger further investigations without clear clinical benefit [3,4]. These challenges have contributed to the growing interest in diagnostic stewardship, an approach aimed at ensuring that diagnostic tests are used appropriately and effectively.

Primary care settings are particularly relevant for the implementation of diagnostic stewardship strategies. Many common presentations encountered in these settings—such as respiratory infections, musculoskeletal complaints, headache, or nonspecific abdominal symptoms—can often be managed through clinical evaluation and basic laboratory testing without immediate imaging. However, variability in clinical judgment, differences in physician experience, and external pressures such as patient expectations may lead to inconsistent imaging practices [5,6].

From a broader perspective, optimizing imaging utilization at the primary care level may have significant system-wide benefits. Reducing unnecessary imaging can lower healthcare costs, decrease cumulative radiation exposure, and minimize the occurrence of incidental findings and subsequent diagnostic cascades. At the same time, it remains essential to ensure timely imaging in conditions where delayed diagnosis may result in adverse outcomes. Therefore, a structured approach that differentiates between situations in which imaging is unnecessary, conditionally indicated, or clearly required may support more balanced and effective diagnostic decision-making [7,8].

Despite increasing attention to diagnostic stewardship, a limited number of studies have attempted to integrate clinical conditions, diagnostic pathways, and system-level outcomes within a unified framework. In this context, simulation-based approaches provide a useful methodological tool. By modeling large populations of hypothetical patient encounters, simulation studies can estimate the potential impact of different diagnostic strategies on imaging utilization, radiation exposure, and healthcare resource consumption without relying on patient-level datasets [9,10].

Primary care encompasses a diverse range of clinical conditions, each associated with varying degrees of diagnostic uncertainty and differing needs for imaging. Organizing these conditions according to their typical diagnostic pathways may provide a practical foundation for guiding imaging utilization. A simplified classification distinguishing between conditions where imaging is generally unnecessary, conditionally indicated, or usually required may help clinicians make more consistent and evidence-informed decisions in routine practice [11,12].

In this context, the present study aimed to develop a simulation-based diagnostic stewardship framework for primary care. A structured dataset consisting of 100 common clinical conditions was constructed, incorporating key variables such as age group distribution, commonly requested laboratory tests, preferred imaging modalities, and imaging necessity classification. Using this dataset, a large-scale simulation model was developed to evaluate the potential impact of a structured diagnostic approach on imaging utilization, radiation burden, incidental diagnostic cascades, and overall resource use.

By integrating perspectives from family medicine, radiology, and public health, this study seeks to provide a conceptual model for improving diagnostic decision-making in primary care. The findings may also inform future efforts to develop clinical decision support tools and policy strategies aimed at promoting more efficient and sustainable use of diagnostic imaging.

## 2. Materials and Methods

### 2.1. Study Design and Conceptual Framework

This study was designed as a simulation-based investigation to develop a conceptual diagnostic stewardship framework for primary care. The model integrates perspectives from family medicine, radiology, and public health to evaluate how structured diagnostic pathways may influence imaging utilization and related outcomes.

A structured dataset consisting of 100 common primary care conditions was constructed to represent a broad spectrum of clinical presentations encountered in routine practice, including respiratory, metabolic, musculoskeletal, neurological, gastrointestinal, genitourinary, dermatologic, psychiatric, cardiopulmonary, and preventive conditions. For each condition, key diagnostic variables were defined, including predominant age group, commonly requested laboratory tests, preferred imaging modality, and imaging necessity classification.

Conditions were categorized into three diagnostic classes: Class A (imaging usually unnecessary), Class B (conditional imaging), and Class C (imaging typically required). This classification reflects typical diagnostic pathways in primary care and serves as the core decision structure of the model.

Additional parameters were incorporated to capture broader implications of imaging decisions, including radiation exposure category, overuse risk, and incidentaloma risk. These variables enabled the model to estimate not only imaging utilization but also downstream consequences such as radiation burden and diagnostic cascades.

A synthetic cohort of one million primary care visits was generated, with case distribution approximating real-world clinical diversity. Imaging decisions were modeled under two scenarios: a baseline scenario reflecting heterogeneous clinical practice and a framework-guided scenario based on the proposed classification system.

Primary outcomes included total imaging utilization, modality distribution, radiation burden index, incidental diagnostic cascades, and a diagnostic cost index. By comparing these scenarios, the study aimed to evaluate the potential system-level impact of structured diagnostic decision-making in primary care.

The use of a synthetic dataset was intentional and aligned with the conceptual objectives of the study. Rather than evaluating an existing clinical intervention, the aim was to explore the potential system-level effects of a structured diagnostic stewardship framework under controlled and transparent conditions. Synthetic data allowed the inclusion of a broad spectrum of primary care conditions within a unified modeling environment and enabled systematic manipulation of imaging utilization parameters that would be difficult to achieve using heterogeneous real-world datasets. Accordingly, the present model should be interpreted as a hypothesis-generating framework designed to support future validation studies rather than as a clinically validated decision-support system.

### 2.2. Construction of the Primary Care Diagnostic Dataset

A structured diagnostic dataset was developed to support the simulation model and to represent the diversity of clinical presentations encountered in primary care. The dataset included 100 common clinical conditions across ten major domains: respiratory, metabolic/endocrine, musculoskeletal, neurological, gastrointestinal, genitourinary, psychiatric, dermatologic, cardiopulmonary, and preventive care. The selection of the 100 clinical conditions was intended to provide broad representation of presentations commonly encountered in routine primary care practice rather than to create an epidemiologically weighted disease registry. Conditions were identified through review of common reasons for primary care consultation reported in the literature, primary care guideline documents, and the clinical expertise of the multidisciplinary author team, which included specialists in family medicine, radiology, and public health. The final dataset was structured to ensure representation across ten major clinical domains relevant to everyday primary care practice. The complete list of included conditions is provided in Supplementary Table S1.

For each condition, key diagnostic variables were defined, including predominant age group (based on World Health Organization classifications), commonly requested laboratory tests, preferred imaging modality, and imaging necessity classification. Imaging modalities included radiography, ultrasound, computed tomography, and magnetic resonance imaging.

Conditions were categorized into three groups according to imaging necessity: Class A (imaging usually unnecessary), Class B (conditional imaging), and Class C (imaging typically required). This classification formed the core structure of the simulation model. Assignment of imaging necessity categories was based on the expected role of imaging within the initial diagnostic evaluation of each condition. Class A included conditions for which imaging is generally unnecessary during routine first-line assessment and where diagnosis is primarily based on clinical evaluation and/or laboratory testing. Class B included conditions in which imaging may be appropriate depending on symptom severity, persistence, treatment response, or the presence of specific clinical warning signs. Class C included conditions in which imaging typically plays a central role in diagnosis, risk stratification, or immediate clinical management.

Risk categories, including radiation exposure, imaging overuse risk, and incidentaloma risk, were assigned using literature-informed assumptions, modality characteristics, and multidisciplinary expert judgment of the author team. These classifications were intended to support comparative simulation modeling rather than to represent formal clinical guideline recommendations.

Additional parameters were incorporated to capture broader diagnostic implications, including radiation exposure category, imaging overuse risk, and incidentaloma risk. These variables enabled estimation of both imaging utilization and downstream outcomes such as radiation burden and diagnostic cascades.

The dataset was organized in a structured tabular format, with each row representing a clinical condition and each column corresponding to a defined diagnostic variable. This structure facilitated integration into the simulation model and enabled systematic analysis of diagnostic pathways across a large synthetic cohort.

Detailed condition-level assignments and associated diagnostic attributes, including imaging necessity classification, radiation exposure category, overuse risk, and incidentaloma risk, are provided in Supplementary Table S2.

The proposed classification system was developed at the condition level and was not intended to provide patient-specific imaging recommendations. Individual imaging decisions in clinical practice may be influenced by numerous patient-level factors, including symptom severity, duration of symptoms, red-flag findings, age, pregnancy status, comorbidities, immunosuppression, prior malignancy, trauma history, neurological deficits, and response to initial management. These factors were intentionally not modeled in the present framework, which was designed as a conceptual representation of diagnostic pathways rather than a patient-level clinical decision-support tool. The classification process was not intended to reproduce any single guideline system (e.g., ACR Appropriateness Criteria, ESR iGuide, or NICE recommendations), but rather to provide a transparent conceptual categorization of imaging necessity suitable for simulation-based modeling.

### 2.3. Simulation Model and Scenario Development

A simulation model was developed to evaluate the impact of the proposed diagnostic stewardship framework on imaging utilization and related outcomes in primary care. The model generated a synthetic cohort of one million primary care visits, with each visit assigned to one of the 100 clinical conditions based on predefined distributions reflecting routine practice.

For each simulated visit, diagnostic attributes from the dataset—including age group, laboratory testing, imaging modality, imaging necessity classification, radiation exposure category, and risk parameters—were applied to approximate clinical decision-making pathways.

Two diagnostic scenarios were modeled. The baseline scenario represented heterogeneous clinical practice, allowing for variable imaging utilization, including potential overuse. The framework-guided scenario applied the structured classification system, reducing imaging in Class A conditions, maintaining conditional use in Class B, and preserving high imaging rates in Class C conditions.

Imaging utilization probabilities were assigned according to imaging necessity class and diagnostic scenario. These probabilities were selected to represent plausible imaging utilization patterns encountered in primary care and to model the expected impact of structured diagnostic stewardship on imaging decision-making. Higher imaging probabilities were permitted in the baseline scenario to reflect heterogeneous clinical practice and potential imaging overuse, whereas lower probabilities were applied in the framework-guided scenario, particularly for Class A conditions. Class B conditions retained intermediate imaging probabilities, while Class C conditions maintained high imaging utilization rates in both scenarios. The complete probability structure used in the simulation model, including imaging utilization probabilities, modality distributions, and sensitivity analysis parameters, is summarized in Supplementary Table S3.

Imaging decisions were determined probabilistically for each scenario, and when performed, the corresponding imaging modality was assigned. This enabled estimation of modality-specific imaging utilization across the simulated population.

In addition to imaging utilization, the model estimated downstream outcomes, including a radiation burden index, incidental diagnostic cascades, and a simplified relative diagnostic resource utilization index. These measures were derived using predefined weighting factors based on imaging modality and cascade probabilities.

Primary outcomes included total imaging examinations, modality distribution, radiation burden, cascade events, and an overall relative diagnostic resource utilization index. These outcomes were compared between the baseline and framework-guided scenarios to assess the system-level impact of the proposed framework.

Imaging decisions were generated using predefined probability parameters assigned to each imaging necessity class. These probabilities were fixed model inputs rather than stochastic Monte Carlo simulations, whereas sensitivity analyses evaluated deterministic variation in these predefined parameters.

### 2.4. Outcome Measures and Analytical Framework

The simulation model was designed to evaluate the impact of the proposed diagnostic stewardship framework on imaging utilization and related outcomes in primary care.

The primary outcome was total imaging utilization, defined as the number of imaging examinations performed in the simulated population. Imaging was further analyzed by modality, including radiography, ultrasound, computed tomography, and magnetic resonance imaging.

Additional outcomes included a radiation burden index, incidental diagnostic cascades, and a relative diagnostic resource utilization index. The radiation burden index was calculated using modality-specific weighting factors based on relative radiation exposure. Incidental cascades were estimated using predefined probabilities of follow-up events triggered by imaging findings. The relative diagnostic resource utilization index was derived from relative weighting factors assigned to imaging modalities and cascade-related investigations. The index was designed as a comparative modeling parameter and does not represent actual healthcare costs or economic outcomes.

Cascade events were modeled as additional diagnostic investigations triggered by incidental imaging findings. Modality-specific cascade probabilities were applied to reflect the likelihood that a given imaging examination would generate further diagnostic work-up, including follow-up imaging or additional clinical evaluation.

The weighting factors and probabilities used for radiation burden estimation, incidental diagnostic cascades, and diagnostic cost calculations were predefined as relative modeling parameters intended to reflect plausible differences among imaging modalities. These values were selected to support comparative simulation analyses and do not represent actual radiation doses, healthcare expenditures, or observed clinical event rates. A detailed summary of all weighting factors and probability assumptions is provided in Supplementary Table S3.

All outcomes were calculated for both the baseline and framework-guided scenarios, allowing direct comparison of imaging utilization and downstream effects between the two approaches.

Analyses were conducted using deterministic parameters defined in the structured dataset and simulation model. As the study was based entirely on simulated data and did not involve human participants, ethical approval was not required.

### 2.5. Sensitivity Analysis and Model Robustness

Sensitivity analyses were performed to assess the robustness of the simulation model under varying assumptions. Key parameters were systematically varied, including baseline imaging utilization probabilities, incidental cascade probabilities, and the distribution of primary care visits across clinical conditions.

Imaging utilization rates were adjusted to reflect scenarios with both higher and lower baseline imaging practices. In addition, cascade probabilities were varied across low, moderate, and high-risk settings, and case-mix distributions were modified to represent different primary care populations.

Across all scenarios, the framework-guided approach consistently resulted in reductions in imaging utilization, radiation burden, and incidental diagnostic cascades. Although the magnitude of these reductions varied, the overall direction of the results remained stable.

Sensitivity analyses were conducted using deterministic parameter variation rather than stochastic Monte Carlo simulations. This approach enabled systematic evaluation of model behavior and supported the robustness of the findings.

### 2.6. Model Implementation and Reproducibility

The simulation framework was implemented using a structured, tabular dataset to ensure transparency and reproducibility. The dataset included 100 primary care conditions, with each row representing a condition and each column corresponding to predefined diagnostic attributes, including age group, laboratory testing, imaging modality, imaging necessity classification, radiation exposure category, and risk parameters.

The model was based on deterministic, rule-based calculations applied to these variables. Each simulated visit was assigned to a clinical condition according to predefined distributions, and imaging decisions were determined under baseline and framework-guided scenarios based on imaging necessity classification.

When imaging was performed, the corresponding modality was assigned, and additional parameters—including radiation weighting, cascade probabilities, and cost indices—were applied to estimate downstream outcomes. A detailed summary of all probability parameters, weighting factors, and simulation assumptions is provided in Supplementary Table S3 to facilitate reproducibility and independent implementation of the framework.

All calculations were conducted using structured spreadsheet-based tools (Microsoft Excel 365; Microsoft Corporation, Redmond, WA, USA), allowing clear documentation of model assumptions and facilitating reproducibility. This framework enables replication and adaptation of the model in different primary care settings. To further enhance reproducibility, all simulation assumptions, imaging utilization probabilities, modality distribution parameters, radiation weighting factors, cascade probabilities, diagnostic cost weights, and sensitivity analysis ranges are summarized in Supplementary Table S3.

### 2.7. Ethical Statement

This study was based entirely on a simulation framework using synthetically generated data and did not involve human participants, patient records, or identifiable personal information. Therefore, ethical approval was not required.

The model was designed to evaluate hypothetical diagnostic pathways and does not represent individual patient outcomes. All parameters and assumptions were defined within a structured computational framework to ensure transparency and reproducibility.

## 3. Results

### 3.1. Characteristics of the Simulated Primary Care Diagnostic Dataset

The diagnostic dataset consisted of 100 clinical conditions representing common primary care presentations, organized across ten domains: respiratory, metabolic/endocrine, musculoskeletal, neurological, gastrointestinal, genitourinary, psychiatric, dermatologic, cardiopulmonary, and preventive care.

Each condition was assigned predefined diagnostic attributes, including World Health Organization age group classification, commonly requested laboratory tests, preferred imaging modality, and imaging necessity classification.

Conditions were categorized into three groups: Class A (imaging usually unnecessary), Class B (conditional imaging), and Class C (imaging usually required). Of the 100 conditions, 63 were classified as Class A, 24 as Class B, and 13 as Class C.

Class A conditions predominated in the psychiatric, dermatologic, metabolic/endocrine, and preventive domains, whereas the highest proportion of Class B conditions was observed in the musculoskeletal domain, followed by the cardiopulmonary and neurological domains. Class C conditions were concentrated in acute or higher-risk clinical presentations in which imaging plays a central diagnostic role.

Among conditions where imaging was considered (Classes B and C), radiography and ultrasound were the most commonly assigned modalities, while computed tomography and magnetic resonance imaging were primarily associated with more complex diagnostic evaluations.

The dataset also incorporated risk parameters, including radiation exposure, imaging overuse risk, and incidentaloma risk, as well as a synthetic distribution of visit frequencies reflecting typical primary care case mix.

**Figure 1:**
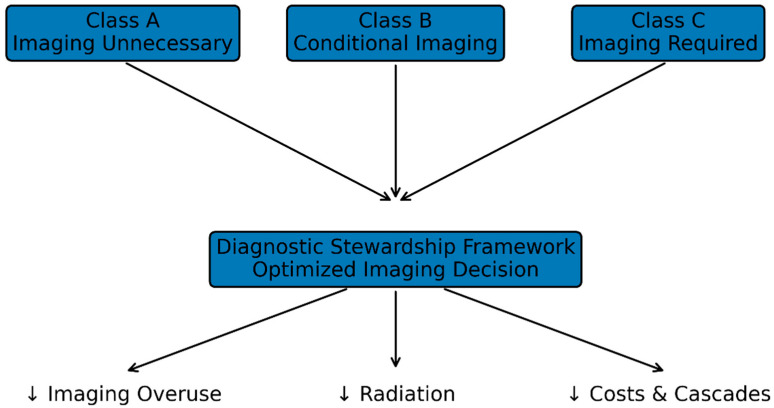
Conceptual overview of the simulation framework showing classification of primary care conditions into Class A, B, and C categories and subsequent modeling of imaging utilization, radiation burden, diagnostic cascades, and resource utilization.

The overall characteristics of the dataset and the distribution of imaging necessity classifications are summarized in Table 1, and the conceptual framework is illustrated in Figure 1.

A schematic diagram illustrating the classification of primary care conditions into three diagnostic pathways (Class A, B, and C), integrating clinical evaluation, laboratory testing, and imaging decision-making processes.

### 3.2. Imaging Utilization in Baseline and Framework Diagnostic Scenarios

The simulation model generated estimates of diagnostic imaging utilization under two scenarios: a baseline diagnostic environment and a framework-guided scenario.

Across one million simulated primary care visits, the baseline scenario produced 412,000 imaging examinations (41.2%), whereas the framework-guided scenario reduced this number to 258,000 (25.8%), corresponding to an overall reduction of approximately 37%.

The reduction in imaging utilization was primarily observed in Class A conditions, where imaging is generally unnecessary during initial primary care evaluation.

Modality-specific analysis demonstrated reductions across all imaging technologies. Conventional radiography was the most frequently used modality in both scenarios, decreasing from 180,000 to 124,000 examinations (31% reduction). Ultrasound examinations decreased from 121,000 to 81,000 (33% reduction).

Computed tomography examinations decreased from 58,000 to 41,000 (29% reduction), while magnetic resonance imaging decreased from 53,000 to 42,000 examinations (21% reduction).

Overall, the simulation model estimated lower imaging utilization across all modalities under the framework-guided scenario while maintaining higher modeled imaging utilization for conditions classified as having greater imaging necessity within the framework.

**Figure 2:**
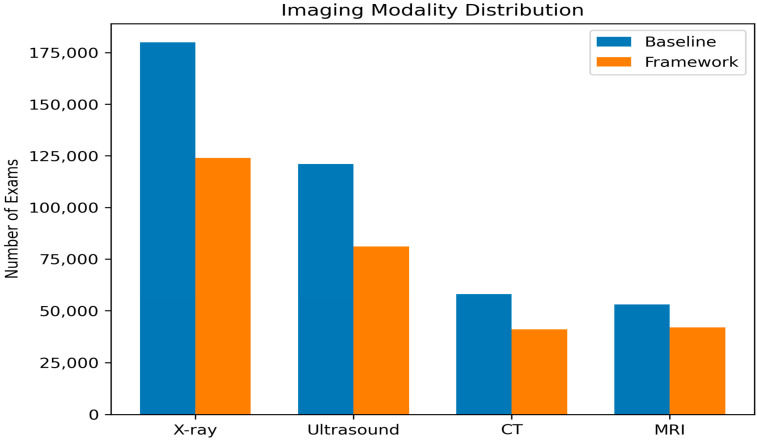
Modality-specific distribution of imaging examinations. Bar chart comparing the number of imaging examinations (X-ray, ultrasound, CT, MRI) between baseline and framework-guided scenarios.

Detailed comparisons of imaging utilization and modality distribution are presented in Table 2 and Figure 2.

### 3.3. Downstream Effects of the Diagnostic Stewardship Framework

In addition to imaging utilization, the simulation model evaluated downstream outcomes, including radiation burden, incidental diagnostic cascades, and diagnostic resource utilization.

The baseline scenario generated a radiation burden index of approximately 285,000 units across one million simulated visits. Under the framework-guided scenario, this value decreased to 179,000 units, corresponding to a reduction of approximately 37%.

The model also estimated incidental diagnostic cascades. In the baseline scenario, approximately 48,200 cascade events were observed, whereas this number decreased to 29,700 under the framework-guided scenario, representing a reduction of approximately 38%.

Diagnostic resource utilization, expressed as a simplified cost index, was estimated at 2,480,000 units in the baseline scenario and 1,690,000 units in the framework-guided scenario, corresponding to a 32% reduction.

Overall, the framework-guided scenario consistently demonstrated reductions across all downstream outcome measures.

**Figure 3:**
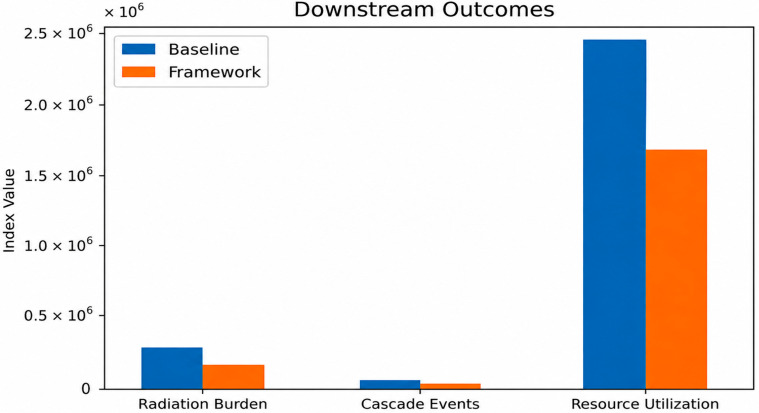
Impact of the diagnostic stewardship framework on downstream outcomes across one million (1 × 10^6^) simulated primary care visits, showing comparative changes in the radiation burden index, diagnostic cascade events, and relative diagnostic resource utilization index between the baseline and framework-guided scenarios.

Detailed comparisons of these outcomes are presented in Table 3 and Figure 3.

### 3.4. Sensitivity Analysis of the Simulation Model

Sensitivity analyses were conducted to evaluate the stability of the simulation outcomes under varying model assumptions.

First, baseline imaging utilization probabilities were adjusted to represent environments with both higher and lower imaging rates. Across these scenarios, the framework-guided approach consistently reduced overall imaging utilization, although the magnitude of reduction varied.

Second, incidental diagnostic cascade probabilities were varied across low-, moderate-, and high-risk settings. While higher cascade probabilities increased follow-up events in the baseline scenario, the framework-guided scenario consistently resulted in fewer cascade events.

Third, the distribution of primary care visits across clinical conditions was modified to reflect different case mix scenarios. Despite these variations, reductions in imaging utilization, radiation burden, and cascade events remained consistent.

Across all sensitivity analyses, the overall direction of results remained stable, with the framework-guided scenario consistently demonstrating lower imaging utilization and downstream effects compared with the baseline scenario.

**Figure 4:**
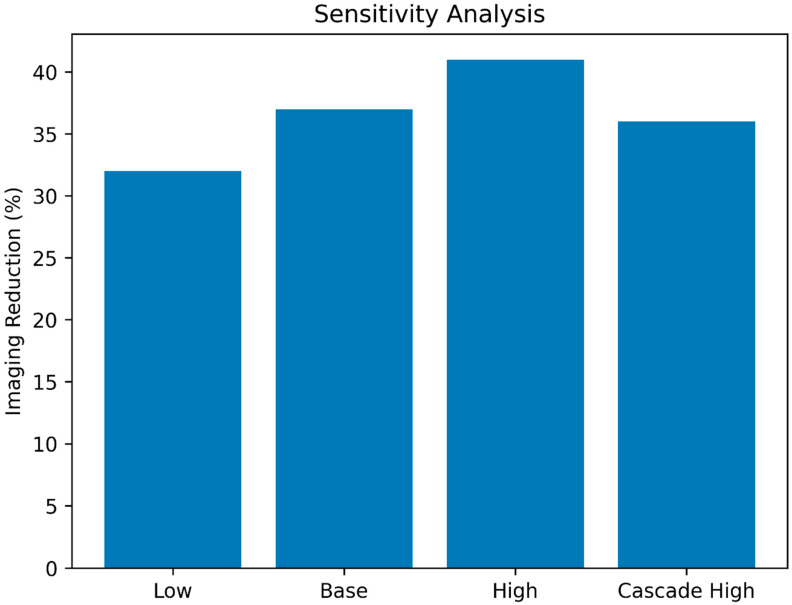
Sensitivity analysis demonstrating the stability of modeled imaging reduction across alternative assumptions for baseline imaging utilization, diagnostic cascade probabilities, and primary care case-mix distributions.

Detailed results are presented in Table 4 and illustrated in Figure 4.

### 3.5. Domain-Specific Effects of the Diagnostic Stewardship Framework

Imaging utilization patterns were analyzed across clinical domains to evaluate the impact of the diagnostic stewardship framework in different clinical contexts.

The magnitude of imaging reduction varied across domains. Domains with a higher proportion of Class A conditions showed the greatest reductions, particularly in psychiatric, dermatologic, respiratory, and preventive care presentations. Domains with a higher proportion of Class B conditions, particularly musculoskeletal, cardiopulmonary, and neurological presentations, demonstrated more moderate reductions, reflecting the conditional role of imaging in these settings.

In contrast, domains containing Class C conditions showed the smallest reductions in imaging utilization, as imaging remains essential for diagnosing high-risk clinical conditions.

Despite these differences, the framework consistently reduced imaging utilization across all domains while maintaining appropriate imaging in clinically indicated scenarios.

Domain-specific analysis also demonstrated variation in modality distribution. Radiography was more frequently used in musculoskeletal and cardiopulmonary conditions, whereas ultrasound was more common in gastrointestinal and genitourinary domains. Advanced imaging modalities, including computed tomography and magnetic resonance imaging, were primarily associated with neurological and abdominal diagnostic pathways.

**Figure 5:**
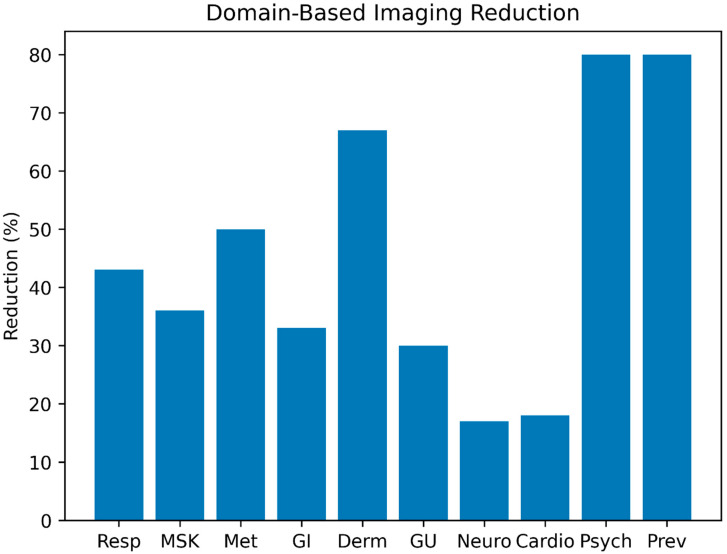
Domain-specific percentage reduction in imaging utilization following implementation of the proposed framework. Imaging reductions ranged from 17% to 80% across clinical domains, with greater reductions observed in domains containing a higher proportion of Class A conditions and smaller reductions in domains where imaging remained clinically indicated.

The distribution of imaging utilization across clinical domains is summarized in Table 5 and illustrated in Figure 5.

It should be noted that the domain-specific baseline imaging rates presented in Table 5 were not determined solely by the distribution of Class A, B, and C conditions shown in Table 1. Rather, they also reflect the baseline imaging probabilities assigned to individual conditions within each class and the simulated case-mix distribution. Consequently, domains with similar class distributions may exhibit different overall imaging rates depending on the underlying probability structure of the simulation model.

## 4. Discussion

### 4.1. Principal Findings

The present simulation-based study developed and evaluated a conceptual diagnostic stewardship framework for primary care by integrating perspectives from family medicine, radiology, and public health. Using a structured dataset representing one hundred common primary care conditions, the simulation model explored how a systematic diagnostic decision framework could influence imaging utilization and downstream healthcare consequences at the population level. The simulation findings suggest that implementation of the proposed framework could generate substantial modeled reductions in unnecessary imaging examinations while maintaining imaging utilization for conditions classified as having higher imaging necessity within the model. These findings are conceptually consistent with the principles of value-based imaging and imaging stewardship, which emphasize optimizing imaging utilization while preserving appropriate diagnostic care [13].

Across the simulated cohort of one million primary care visits, the simulation estimated a marked reduction in imaging utilization, with total imaging examinations decreasing by approximately one third compared with the baseline diagnostic scenario. This reduction was primarily driven by decreased imaging in clinical situations where imaging is generally unnecessary during the initial diagnostic evaluation. In many routine primary care encounters, particularly those involving mild respiratory infections, dermatologic complaints, or mental health consultations, diagnostic imaging rarely contributes meaningfully to clinical decision-making. The structured classification system introduced in the framework enabled these low-risk clinical situations to be managed primarily through clinical assessment and selective laboratory evaluation [14].

Importantly, the reduction in imaging utilization did not occur uniformly across all clinical domains. The domain-specific analysis demonstrated that the framework achieved the largest reductions in imaging for clinical presentations with a high proportion of conditions categorized as Class A, where imaging is rarely required. In contrast, domains containing conditions where imaging plays a central diagnostic role—such as neurological and certain cardiopulmonary presentations—showed smaller reductions in imaging utilization. This pattern suggests that the framework may preferentially allocate imaging to conditions classified as having greater imaging necessity within the simulation model, rather than uniformly reducing imaging across all clinical scenarios [15].

Beyond the reduction in imaging examinations, the simulation results also demonstrated meaningful system-level benefits related to radiation exposure and downstream diagnostic consequences. The simulation estimated a lower population-level radiation burden under the framework-guided scenario following implementation of the framework. This reduction primarily reflects the decreased use of radiography and computed tomography examinations in low-risk clinical scenarios. From a public health perspective, reductions in unnecessary radiation exposure are particularly relevant given the cumulative nature of radiation exposure across populations and the increasing global utilization of diagnostic imaging technologies [16].

Another important finding of the simulation was the reduction in incidental diagnostic cascades triggered by imaging examinations. Incidental findings are a well-recognized consequence of modern imaging technologies and frequently lead to additional diagnostic investigations, follow-up imaging, and specialist referrals. While some incidental findings may ultimately reveal clinically important conditions, many represent benign or clinically insignificant abnormalities that nevertheless initiate further diagnostic evaluation. The simulation suggests that modeled reductions in imaging utilization may decrease the number of incidental findings leading to downstream cascade events within the assumptions of the framework [17]. It should be noted, however, that incidental finding rates vary considerably across imaging modalities, anatomical regions, and clinical contexts. In the present framework, cascade probabilities were modeled using simplified risk categories intended to represent downstream diagnostic consequences at a system level rather than condition-specific incidentaloma rates. Therefore, the observed reduction in cascade events should be interpreted primarily as an illustration of how lower imaging utilization may influence downstream diagnostic activity within the conceptual model.

In addition to reductions in radiation burden and cascade events, the framework was associated with a notable decrease in the overall diagnostic resource utilization index. Because advanced imaging modalities such as computed tomography and magnetic resonance imaging typically require greater healthcare resources than basic imaging examinations, reductions in unnecessary imaging were estimated to contribute to more efficient utilization of diagnostic resources. Although the present model used a simplified relative diagnostic resource utilization index rather than real-world economic data, this index was intended solely as a comparative modeling parameter and should not be interpreted as a measure of actual healthcare costs or economic benefit. These findings suggest the potential for more efficient diagnostic resource use under the assumptions of the simulation model [18].

The robustness of these findings was further supported by the sensitivity analyses conducted within the simulation model. Across multiple alternative parameter scenarios—including variations in baseline imaging utilization rates, incidental cascade probabilities, and primary care case mix distributions—the framework consistently demonstrated reductions in imaging utilization and downstream diagnostic consequences. These results suggest that the proposed the modeled effects remained robust across the tested parameter assumptions [19]. Importantly, the sensitivity analyses demonstrated that the framework’s overall direction of effect remained consistent despite variations in baseline imaging utilization, cascade probabilities, and case-mix distributions. Although the magnitude of benefit varied across scenarios, reductions in imaging utilization, radiation burden, and cascade events were observed in all tested conditions. This finding suggests that the modeled effects were not driven by a single parameter assumption and supports the internal robustness of the conceptual framework.

Taken together, the findings of this simulation study suggest that structured diagnostic stewardship strategies may offer a promising approach for optimizing imaging utilization in primary care. By combining clinical decision pathways with structured diagnostic classification, the framework illustrates how diagnostic imaging can be used more selectively while maintaining appropriate access for patients with potentially serious conditions.

### 4.2. Diagnostic Stewardship in Primary Care

Primary care plays a pivotal role in shaping diagnostic pathways within healthcare systems. As the first point of contact for most patients, family physicians evaluate a wide spectrum of conditions, from minor self-limiting illnesses to potentially life-threatening diseases. Consequently, diagnostic decisions made in primary care strongly influence downstream utilization of laboratory tests, imaging, specialist referrals, and hospital-based services, with important implications for healthcare efficiency and patient safety [20].

The concept of diagnostic stewardship has gained increasing attention as healthcare systems seek to balance the benefits of advanced diagnostic technologies with the risks of overutilization. It refers to ensuring that the right test is performed for the right patient at the right time. While widely applied to laboratory testing, its role in imaging utilization within primary care remains relatively underexplored [21].

The findings of the present simulation study suggest that structured diagnostic frameworks may provide a practical approach for implementing diagnostic stewardship in primary care. By categorizing clinical conditions according to their typical diagnostic pathways, the proposed framework offers a structured decision-support approach that may help clinicians differentiate between situations where imaging is unnecessary, conditionally indicated, or essential for diagnosis [22].

Such frameworks may be particularly valuable in situations characterized by diagnostic uncertainty, which is common in primary care. Patients frequently present with nonspecific symptoms, where imaging decisions may vary depending on physician experience, patient expectations, and concerns about missed diagnoses. Structured decision pathways may help reduce this variability while maintaining patient safety [23].

Diagnostic stewardship is also closely linked to the gatekeeping role of primary care physicians. In many healthcare systems, imaging requests originating from primary care represent a substantial portion of overall imaging demand. Therefore, improving diagnostic decision-making at this stage may have significant downstream effects on healthcare utilization [24].

The proposed framework aligns with value-based healthcare principles by promoting selective imaging based on clinical context. This approach may contribute to more efficient resource utilization while preserving the diagnostic benefits of advanced imaging modalities [25].

Furthermore, integrating radiology perspectives into primary care decision-making may enhance interdisciplinary collaboration. Improved communication between primary care providers and radiologists, supported by structured frameworks, may help ensure that imaging studies are requested in clinically appropriate situations [26].

Overall, diagnostic stewardship in primary care represents an important opportunity to improve the efficiency and safety of diagnostic processes. The results of this simulation study illustrate how structured diagnostic pathways may support this objective by generating modeled reductions in imaging utilization while maintaining higher modeled imaging use for conditions classified as having greater imaging necessity within the framework [27].

### 4.3. Public Health Implications of Imaging Optimization

Beyond its clinical relevance, optimizing diagnostic imaging utilization in primary care carries important public health implications. As imaging technologies become increasingly accessible, the cumulative impact of imaging decisions at the population level has become a growing concern. The findings of this simulation study suggest that structured diagnostic stewardship strategies may improve diagnostic resource utilization within the assumptions of the model and warrant evaluation in real-world healthcare settings [28].

One of the most important considerations is cumulative radiation exposure. Imaging modalities involving ionizing radiation, particularly computed tomography and conventional radiography, contribute to the overall radiation burden. Although individual examinations involve relatively low doses, repeated imaging across large populations may lead to substantial cumulative exposure. Even modest reductions in unnecessary imaging may therefore translate into meaningful decreases in population-level radiation exposure [29].

In the present simulation, implementation of the diagnostic stewardship framework resulted in a substantial reduction in the estimated radiation burden index. This reduction was primarily driven by decreased imaging in low-risk clinical conditions where imaging provides limited diagnostic benefit. Given the central role of primary care in initial diagnostic evaluation, such improvements may have broad public health implications [30].

Another important consideration is the occurrence of incidental findings. Incidentalomas may trigger additional diagnostic investigations, follow-up imaging, and specialist referrals. While some findings may be clinically relevant, many are benign or insignificant, yet still lead to further testing [31].

At the population level, these cascade events may increase healthcare utilization and patient burden. The reduction in cascade events observed in this simulation suggests that decreasing unnecessary imaging may also reduce the frequency of incidental findings that lead to additional investigations [32].

Imaging optimization also has implications for healthcare system sustainability. Advanced imaging modalities contribute significantly to healthcare expenditures, and strategies promoting appropriate imaging use may help ensure that diagnostic resources remain available for patients who require them most [33].

Furthermore, structured diagnostic frameworks align with broader public health goals of evidence-based resource utilization. By supporting more selective imaging decisions, such approaches may contribute to more efficient and sustainable healthcare systems [34].

From a broader perspective, improving diagnostic stewardship in primary care may also help reduce disparities in healthcare access by preserving imaging capacity for high-risk patients.

Overall, these findings illustrate the potential role of diagnostic stewardship as a conceptual public health strategy that may support more appropriate imaging utilization within the assumptions of the simulation model and warrants future validation in real-world clinical settings.

### 4.4. Comparison with Existing Literature

The increasing utilization of diagnostic imaging has been widely discussed in the medical literature, particularly in relation to imaging overuse and its potential consequences for healthcare systems and patient safety. Numerous studies have reported substantial growth in the use of imaging technologies over recent decades, especially for computed tomography and magnetic resonance imaging. This expansion has been attributed to technological advancements, broader clinical indications, increased availability of imaging equipment, and evolving expectations from clinicians and patients [35].

Within this context, appropriate imaging utilization has become an important focus of healthcare policy and clinical research. Several international initiatives have addressed unnecessary imaging through clinical guidelines and decision-support tools. One of the most recognized efforts is the Choosing Wisely initiative, which encourages physicians to avoid tests that are unlikely to provide meaningful clinical benefit, including imaging in low-risk clinical scenarios [36].

Similarly, radiology has increasingly emphasized imaging stewardship, paralleling antimicrobial stewardship principles. Imaging stewardship promotes the use of imaging only in situations where results are likely to influence patient management. Radiology societies have therefore supported clinical decision support systems, appropriateness criteria, and educational interventions to guide evidence-based imaging practices [37].

Despite these efforts, implementing appropriate imaging utilization in routine practice remains challenging, particularly in primary care. Physicians frequently encounter patients with nonspecific symptoms, where imaging decisions may be influenced by diagnostic uncertainty, time constraints, patient expectations, and medicolegal concerns. As a result, variability in imaging utilization persists across clinical settings [38].

The simulation-based framework proposed in this study contributes to this field by providing a conceptual model tailored to primary care. Unlike traditional guidelines focused on individual conditions, this framework categorizes a wide range of primary care presentations according to their diagnostic pathways, allowing evaluation across diverse clinical scenarios [39].

Another important contribution is the use of simulation modeling to explore system-level consequences of diagnostic decision-making. While previous studies often rely on retrospective datasets, simulation models allow the assessment of hypothetical diagnostic strategies and their potential impact prior to clinical implementation [40].

The findings of this study are consistent with previous research suggesting that a substantial proportion of imaging examinations may have limited diagnostic value, while also reinforcing the need to maintain imaging access for clinically significant conditions. The framework therefore aligns with the central principle of imaging stewardship: optimizing imaging utilization rather than reducing it indiscriminately.

Overall, these results extend the existing literature by demonstrating how structured diagnostic classification systems may help operationalize imaging stewardship principles within primary care. By integrating clinical context, imaging necessity classification, and simulation modeling, the proposed framework offers a complementary perspective to guideline-based approaches.

### 4.5. Strengths and Limitations

The present study has several strengths that contribute to its methodological and conceptual relevance. First, the study introduces a structured diagnostic stewardship framework specifically designed for primary care environments, integrating perspectives from family medicine, radiology, and public health. Primary care represents a complex diagnostic environment where clinicians must evaluate a wide range of clinical presentations with varying levels of diagnostic uncertainty. By constructing a dataset that includes one hundred common primary care conditions across multiple clinical domains, the study provides a broad conceptual representation of diagnostic decision-making in routine primary care practice.

Second, the study employs a simulation-based methodological approach, which allows the evaluation of hypothetical diagnostic strategies without relying on patient-level clinical data. Simulation modeling offers a valuable tool for exploring the potential system-level consequences of diagnostic decisions before implementing such strategies in real clinical environments. By modeling one million simulated primary care visits, the framework was able to generate stable estimates of imaging utilization, radiation burden, incidental diagnostic cascades, and diagnostic resource consumption. This large-scale modeling approach enabled the analysis of diagnostic patterns at a population level, which would be difficult to achieve using conventional clinical datasets.

Another strength of the study is the incorporation of multiple dimensions of diagnostic outcomes, including imaging utilization, modality distribution, radiation burden, incidental cascade events, and an overall diagnostic cost index. Many previous studies examining imaging utilization have focused primarily on imaging frequency alone. By integrating these additional outcome measures, the present model provides a more comprehensive assessment of the potential consequences of diagnostic decision-making in primary care.

Furthermore, the inclusion of sensitivity analyses strengthens the internal consistency of the simulation model. By testing the model under alternative assumptions regarding imaging utilization probabilities, cascade event probabilities, and case mix distributions, the study demonstrated that the general pattern of results remained stable across a range of plausible scenarios. This robustness suggests that the conceptual framework may retain its potential effectiveness across different healthcare environments and diagnostic practice patterns.

Despite these strengths, several limitations should also be acknowledged. First, the study is based on a synthetic simulation model rather than real-world clinical data. Although the simulation framework was designed to approximate common diagnostic pathways encountered in primary care practice, it cannot fully capture the complexity and variability of real patient encounters. Factors such as physician experience, patient expectations, local healthcare policies, and resource availability may influence imaging decisions in ways that are difficult to represent within a simplified simulation environment. Furthermore, the model was not calibrated against a real-world primary care dataset. The purpose of the present study was to establish a conceptual and reproducible framework for evaluating diagnostic stewardship strategies rather than to generate precise estimates of clinical practice patterns. Consequently, the reported outcomes should be interpreted as illustrative model outputs under predefined assumptions. Future studies incorporating electronic health record data, imaging utilization databases, or prospective primary care cohorts will be necessary to calibrate model parameters and evaluate the external validity of the framework.

Second, the diagnostic dataset used in the model represents a conceptual representation of common primary care conditions rather than an empirically derived epidemiological dataset. While the conditions included in the dataset reflect typical primary care presentations, the relative frequency of these conditions and their associated diagnostic pathways may vary across different healthcare systems and geographic regions.

Another limitation relates to the simplified diagnostic resource utilization and radiation burden indices used in the simulation. These indices were designed to provide comparative estimates of diagnostic resource utilization rather than precise measurements of healthcare expenditures or radiation doses. Real-world economic analyses and dosimetric calculations would require more detailed clinical data and healthcare system–specific cost information.

In addition, the sensitivity analyses focused on plausible parameter variations around the predefined base-case assumptions. More extreme parameter settings, including healthcare environments characterized by substantially higher baseline imaging utilization, were not explicitly modeled and may be explored in future studies.

Another limitation relates to the simplified modeling of incidental diagnostic cascades. Cascade probabilities were assigned using broad literature-informed risk categories and expert consensus rather than modality-specific or condition-specific empirical estimates. Consequently, reductions in cascade events were influenced predominantly by changes in imaging utilization rather than by detailed modeling of incidental finding behavior across different anatomical regions or imaging modalities. Future studies may incorporate modality-specific, organ-specific, and condition-specific incidentaloma rates derived from real-world datasets to provide a more granular representation of downstream diagnostic pathways.

Finally, the proposed diagnostic stewardship framework represents a conceptual model rather than a clinical decision support tool currently implemented in practice. Further research will therefore be necessary to evaluate how similar frameworks might be integrated into real clinical workflows, potentially through electronic decision-support systems or clinical guideline development.

Despite these limitations, the present study provides an illustrative model demonstrating how structured diagnostic frameworks may influence imaging utilization and downstream healthcare outcomes. The results highlight the potential value of simulation-based approaches for exploring diagnostic stewardship strategies and may serve as a foundation for future empirical studies evaluating diagnostic optimization in primary care settings.

### 4.6. Novelty and Contribution of the Study

The present study introduces a novel conceptual approach to diagnostic stewardship in primary care by integrating clinical decision pathways, imaging utilization patterns, and population-level simulation modeling within a single analytical framework. While previous studies have examined imaging overuse or appropriateness criteria in specific clinical contexts, relatively few investigations have attempted to model diagnostic decision-making across a broad spectrum of primary care conditions.

A distinctive aspect of this study is the development of a structured diagnostic dataset consisting of one hundred common primary care conditions, organized across multiple clinical domains and linked to diagnostic attributes such as laboratory testing, preferred imaging modalities, and imaging necessity classifications. This dataset enabled the construction of a simulation model capable of representing the heterogeneous diagnostic landscape encountered in primary care practice.

Another novel contribution of this work is the integration of multiple system-level outcome measures within a single simulation framework. Rather than focusing solely on imaging utilization rates, the model simultaneously evaluates population-level radiation burden, incidental diagnostic cascades, and diagnostic resource consumption. This multidimensional approach provides a broader perspective on the potential consequences of diagnostic decision-making.

Furthermore, the study demonstrates how simulation-based modeling can be used as a methodological tool for exploring diagnostic stewardship strategies in healthcare systems where empirical patient-level datasets may be limited or unavailable. By generating a large synthetic cohort of primary care visits, the framework allows researchers to evaluate potential diagnostic policies and clinical decision pathways before their implementation in real clinical environments.

Taken together, these elements position the present study as an exploratory step toward the development of structured diagnostic stewardship frameworks that integrate primary care, radiology, and public health perspectives. The proposed approach may serve as a conceptual foundation for future research aimed at developing decision-support tools, clinical guidelines, or health system strategies designed to optimize diagnostic imaging utilization.

### 4.7. Future Research Directions

The conceptual framework presented in this study represents an initial step toward the development of structured diagnostic stewardship strategies for primary care. While the simulation results provide insights into potential system-level effects, further research is needed to validate and expand this approach.

An important priority for future research will be external validation of the framework using real-world primary care imaging utilization datasets. Such validation studies will be necessary to assess the generalizability of the proposed classification system and to determine whether the patterns observed in the simulation model are reproducible in routine clinical practice.

First, future studies should evaluate the clinical applicability of the framework using real-world primary care datasets. Large-scale observational studies incorporating electronic health records may provide empirical evidence on imaging utilization patterns and allow comparison with the simulated model.

Second, the framework could be developed into a clinical decision-support tool integrated within electronic health record systems. Such tools may assist clinicians in determining when imaging is appropriate, conditionally indicated, or unnecessary, potentially reducing variability in diagnostic decision-making while preserving clinical judgment.

Another important direction involves the prospective evaluation of diagnostic stewardship interventions in primary care. Pilot implementation studies could assess whether structured frameworks influence imaging ordering behavior and improve healthcare efficiency without compromising diagnostic accuracy or patient outcomes.

In addition, future research may explore interactions between diagnostic stewardship frameworks and emerging technologies, including artificial intelligence–based decision-support systems. Machine learning models may help generate individualized recommendations for imaging utilization based on patient characteristics and clinical presentation.

Finally, further studies should examine the economic implications of imaging optimization strategies. Cost-effectiveness analyses using real-world data may provide valuable insights into the potential financial benefits of reducing unnecessary imaging and diagnostic cascades.

Future methodological extensions may also incorporate probabilistic sensitivity analyses and multivariate uncertainty modeling. Such approaches could evaluate the simultaneous effects of parameter variability and interactions among model assumptions, providing a more comprehensive assessment of uncertainty and model robustness than deterministic sensitivity analyses alone.

Overall, integrating empirical data, decision-support tools, and prospective implementation studies will be essential for translating this conceptual framework into practical diagnostic stewardship strategies in primary care.

## 5. Conclusions

The present study proposes a conceptual diagnostic stewardship framework designed to optimize imaging utilization in primary care settings by integrating perspectives from family medicine, radiology, and public health. Using a structured dataset representing one hundred common primary care conditions and a large-scale simulation model of one million hypothetical primary care visits, the study explored how structured diagnostic decision pathways may influence imaging utilization and downstream healthcare consequences at the population level.

The simulation results suggest that the implementation of a structured diagnostic framework may lead to substantial reductions in unnecessary imaging examinations while maintaining appropriate access to diagnostic imaging for conditions in which imaging plays a critical role in patient evaluation. By categorizing clinical conditions according to their typical diagnostic pathways, the framework enables clinicians to differentiate between situations where imaging is generally unnecessary, conditionally indicated, or essential for diagnosis. This structured approach may help reduce variability in diagnostic decision-making while preserving clinical flexibility in complex cases.

Beyond reducing imaging utilization, the framework also demonstrated potential system-level benefits, including reductions in population-level radiation exposure, incidental diagnostic cascades, and overall diagnostic resource consumption. These findings highlight the broader implications of imaging optimization strategies not only for individual patient care but also for healthcare system efficiency and sustainability. Given the growing demand for diagnostic imaging services worldwide, strategies that promote appropriate imaging utilization are likely to become increasingly important in maintaining the balance between technological advancement and responsible resource management.

From a public health perspective, improving diagnostic stewardship at the primary care level may represent a particularly impactful strategy. Because primary care physicians serve as the initial point of diagnostic evaluation for a large proportion of patients, optimizing imaging decisions at this stage of the healthcare pathway may have widespread effects on downstream healthcare utilization. The framework presented in this study illustrates how structured diagnostic approaches may support clinicians in navigating diagnostic uncertainty while minimizing unnecessary diagnostic interventions.

Although the present study is based on a simulation model rather than empirical clinical data, the findings provide an illustrative demonstration of how structured diagnostic frameworks may influence imaging utilization patterns in primary care environments. Future research incorporating real-world clinical datasets and prospective implementation studies will be essential to evaluate the practical feasibility and clinical effectiveness of similar diagnostic stewardship strategies.

In conclusion, the results of this simulation study suggest that structured diagnostic stewardship frameworks may provide a conceptual approach for optimizing imaging utilization in primary care. Within the assumptions of the simulation model, the proposed framework generated modeled reductions in imaging utilization, radiation burden, and downstream diagnostic consequences. These findings are hypothesis-generating and require validation using real-world patient-level data before any clinical application, decision-support implementation, or healthcare policy implications can be inferred.

## Figures and Tables

**Table 1:** Characteristics of the simulated diagnostic dataset and imaging necessity distribution across clinical domains.

Clinical Domain	Number of Conditions	Class A	Class B	Class C
Respiratory	10	6	2	2
Metabolic/Endocrine	10	8	2	0
Musculoskeletal	10	4	6	0
Neurological	10	5	3	2
Gastrointestinal	10	7	2	1
Genitourinary	10	5	2	3
Psychiatric	10	9	1	0
Dermatologic	10	9	1	0
Cardiopulmonary	10	3	4	3
Preventive	10	7	1	2
Total	100	63	24	13

**Table 2:** Imaging utilization and modality distribution in baseline and framework-guided scenarios.

Parameter	Baseline	Framework	Reduction (%)
Total imaging exams	412,000	258,000	37%
Imaging rate (%)	41.2%	25.8%	—
X-ray	180,000	124,000	31%
Ultrasound	121,000	81,000	33%
CT	58,000	41,000	29%
MRI	53,000	42,000	21%

**Table 3:** Downstream diagnostic outcomes in baseline vs. framework scenarios.

Outcome	Baseline	Framework	Reduction (%)
Radiation burden index	285,000	179,000	37%
Cascade events	48,200	29,700	38%
**Relative diagnostic resource utilization index**	2,480,000	1,690,000	32%

**Table 4:** Sensitivity analysis results across alternative simulation scenarios.

Scenario	Imaging Reduction	Radiation Reduction	Cascade Reduction
Low imaging baseline	32%	30%	31%
Base model	37%	37%	38%
High imaging baseline	41%	39%	42%
High cascade probability	36%	37%	45%

**Table 5:** Domain-specific imaging utilization before and after framework implementation.

Clinical Domain	Baseline Imaging (%)	Framework Imaging (%)	Reduction (%)
Respiratory	35	20	43%
Musculoskeletal	50	32	36%
Metabolic/Endocrine	20	10	50%
Gastrointestinal	45	30	33%
Dermatologic	15	5	67%
Genitourinary	40	28	30%
Neurological	60	50	17%
Cardiopulmonary	55	45	18%
Psychiatric	10	2	80%
Preventive	5	1	80%

## Data Availability

The datasets generated and analyzed during the current study consist entirely of synthetic data produced within a computational simulation framework. No real patient data, medical images, or identifiable information were used. The simulation dataset and modeling parameters supporting the findings of this study are available from the corresponding author upon reasonable request.

## Supplementary Materials

The following supporting information can be downloaded at: https://www.mdpi.com/article/10.3390/diagnostics16142162/s1. Supplementary Table S1. Complete List of Primary Care Conditions and Clinical Domain Classification. Supplementary Table S2. Diagnostic attributes associated with each clinical condition. Includes age group classification, commonly requested laboratory tests, preferred imaging modality, imaging necessity class (A–C), radiation exposure category, and incidentaloma risk classification. Supplementary Table S3. Simulation Model Parameters. Supplementary File S1. Structured dataset used for the simulation model, Complete diagnostic framework with clinical conditions, diagnostic attributes, and linked simulation parameter definitions.
