# Supplementary material for: A Simulation-Based Diagnostic Stewardship Framework for Imaging Utilization in Primary Care: A Model Using 100 Common Clinical Conditions
Source: Diagnostics (Basel). 2026 Jul 10;16(14):2162. doi: 10.3390/diagnostics16142162 (PMC13409685; doi:10.3390/diagnostics16142162)
Supplement: Supplementary file 1 [file diagnostics-16-02162-s001.zip › Supplementary Table S1-A Simulation-Based Diagnostic Stewardship Framework for Imaging Utilization in Primary Care.pdf]

Supplementary Table S1. Complete List of Primary Care Conditions and Clinical Domain Classification

No	Clinical Condition	Clinical Domain
1	Acute upper respiratory tract infection	Respiratory
2	Acute bronchitis	Respiratory
3	Community-acquired pneumonia	Respiratory
4	Asthma exacerbation	Respiratory
5	Chronic obstructive pulmonary disease (COPD) exacerbation	Respiratory
6	Allergic rhinitis	Respiratory
7	Sinusitis	Respiratory
8	Influenza	Respiratory
9	COVID-19 infection	Respiratory
10	Chronic cough	Respiratory
11	Type 2 diabetes mellitus	Metabolic/Endocrine
12	Hypertension	Metabolic/Endocrine
13	Hypothyroidism	Metabolic/Endocrine
14	Hyperthyroidism	Metabolic/Endocrine
15	Dyslipidemia	Metabolic/Endocrine
16	Obesity	Metabolic/Endocrine
17	Metabolic syndrome	Metabolic/Endocrine
18	Vitamin D deficiency	Metabolic/Endocrine
19	Anemia (nutritional)	Metabolic/Endocrine
20	Osteoporosis	Metabolic/Endocrine
21	Low back pain	Musculoskeletal
22	Neck pain	Musculoskeletal
23	Knee osteoarthritis	Musculoskeletal
24	Shoulder impingement syndrome	Musculoskeletal
25	Muscle strain	Musculoskeletal
26	Tendinitis	Musculoskeletal
27	Carpal tunnel syndrome	Musculoskeletal
28	Sciatica	Musculoskeletal
29	Joint sprain	Musculoskeletal
30	Fibromyalgia	Musculoskeletal
31	Tension-type headache	Neurological
32	Migraine	Neurological
33	Dizziness/vertigo	Neurological

No	Clinical Condition	Clinical Domain
34	Peripheral neuropathy	Neurological
35	Syncope	Neurological
36	Seizure (first episode)	Neurological
37	Suspected stroke	Neurological
38	Bell's palsy	Neurological
39	Memory impairment	Neurological
40	Tremor	Neurological
41	Acute gastroenteritis	Gastrointestinal
42	Gastroesophageal reflux disease (GERD)	Gastrointestinal
43	Irritable bowel syndrome (IBS)	Gastrointestinal
44	Constipation	Gastrointestinal
45	Acute abdominal pain	Gastrointestinal
46	Gallstone disease	Gastrointestinal
47	Peptic ulcer disease	Gastrointestinal
48	Hepatitis	Gastrointestinal
49	Appendicitis (suspected)	Gastrointestinal
50	Hemorrhoids	Gastrointestinal
51	Urinary tract infection (UTI)	Genitourinary
52	Acute pyelonephritis	Genitourinary
53	Renal colic (urolithiasis)	Genitourinary
54	Benign prostatic hyperplasia	Genitourinary
55	Hematuria	Genitourinary
56	Urinary incontinence	Genitourinary
57	Vaginitis	Genitourinary
58	Pelvic pain	Genitourinary
59	Erectile dysfunction	Genitourinary
60	Testicular pain	Genitourinary
61	Depression	Psychiatric
62	Anxiety disorder	Psychiatric
63	Panic disorder	Psychiatric
64	Insomnia	Psychiatric
65	Somatization disorder	Psychiatric
66	Adjustment disorder	Psychiatric
67	Substance use disorder	Psychiatric
68	Burnout/stress-related disorder	Psychiatric
69	Attention deficit symptoms	Psychiatric
70	Mild cognitive disorder	Psychiatric
71	Dermatitis (eczema)	Dermatologic
72	Acne vulgaris	Dermatologic
73	Psoriasis	Dermatologic

No	Clinical Condition	Clinical Domain
74	Skin infection (cellulitis)	Dermatologic
75	Fungal skin infection	Dermatologic
76	Urticaria	Dermatologic
77	Skin lesion (benign nevus)	Dermatologic
78	Suspicious skin lesion	Dermatologic
79	Alopecia	Dermatologic
80	Nail disorders	Dermatologic
81	Chest pain	Cardiopulmonary
82	Palpitations	Cardiopulmonary
83	Dyspnea	Cardiopulmonary
84	Suspected heart failure	Cardiopulmonary
85	Deep vein thrombosis (suspected)	Cardiopulmonary
86	Pulmonary embolism (suspected)	Cardiopulmonary
87	Peripheral edema	Cardiopulmonary
88	Syncope (cardiac origin)	Cardiopulmonary
89	Arrhythmia	Cardiopulmonary
90	Hypertensive crisis	Cardiopulmonary
91	Routine health check-up	Preventive
92	Cancer screening visit	Preventive
93	Vaccination visit	Preventive
94	Prenatal care visit	Preventive
95	Postnatal care visit	Preventive
96	Growth and development assessment	Preventive
97	Smoking cessation counseling	Preventive
98	Weight management consultation	Preventive
99	Occupational health evaluation	Preventive
100	General fatigue evaluation	Preventive
