# Supplementary material for: A Simulation-Based Diagnostic Stewardship Framework for Imaging Utilization in Primary Care: A Model Using 100 Common Clinical Conditions
Source: Diagnostics (Basel). 2026 Jul 10;16(14):2162. doi: 10.3390/diagnostics16142162 (PMC13409685; doi:10.3390/diagnostics16142162)
Supplement: Supplementary file 1 [file diagnostics-16-02162-s001.zip › Supplementary Table S3-A Simulation-Based Diagnostic Stewardship Framework for Imaging Utilization in Primary Care.pdf]

Supplementary Table S3. Simulation Model Parameters

A. Imaging Utilization Probabilities by Diagnostic Class

Imaging Class	Description	Baseline Scenario Probability	Framework Scenario Probability
Class A	Imaging usually unnecessary	0.30	0.05
Class B	Conditional imaging	0.60	0.40
Class C	Imaging usually required	0.90	0.85

B. Imaging Modality Distribution Probabilities (if imaging performed)

Modality	Baseline Probability	Framework Probability	Radiation Category
X-ray	0.44	0.48	Low
Ultrasound	0.29	0.31	None
CT	0.14	0.12	Medium
MRI	0.13	0.09	None

✓ Adjusted to reflect:

- Slight CT/MRI reduction in framework
- Increased reliance on US/X-ray (primary care realistic)

C. Incidental Diagnostic Cascade Probabilities

Incidentaloma Risk	Description	Cascade Probability
Low	Minimal incidental findings	0.05
Medium	Occasional incidental findings	0.15
High	Frequent incidental findings	0.30

D. Radiation Burden Weighting Factors

Imaging Modality	Relative Radiation Weight
None	0
Ultrasound	0
MRI	0
X-ray	1
CT	5

✓ Reflects standard radiological hierarchy

E. Diagnostic Cost Index Weighting Factors

Component	Weight
X-ray	1
Ultrasound	2
CT	5
MRI	6
Cascade follow-up event	3

F. Primary Care Visit Distribution (Model Input)

Clinical Domain	Proportion (%)
Respiratory	18
Musculoskeletal	16
Metabolic/Endocrine	12
Gastrointestinal	12
Dermatologic	10
Genitourinary	8
Neurological	8
Cardiopulmonary	8
Psychiatric	5
Preventive	3

G. Sensitivity Analysis Ranges

Parameter	Low	Base	High
Class A imaging probability	0.20	0.30	0.40

Parameter	Low	Base	High
Class B imaging probability	0.50	0.60	0.70
Cascade probability (Medium risk)	0.10	0.15	0.20
CT usage proportion	0.10	0.14	0.18
